# Supplementary material for: Community perception differences regarding ecosystem goods and services in reservoir landscapes with and without inter-basin water transfer: Implications for the Sustainable Development Goals
Source: PLoS One. 2025 May 20;20(5):e0323670. doi: 10.1371/journal.pone.0323670 (PMC12091769; doi:10.1371/journal.pone.0323670)
Supplement: Attachment — Submitted filename: PlOS One second reviews.pdf [file pone.0323670.s005.pdf]

PlOS One second reviews

Title: Community perception differences regarding of ecosystem goods and services in landscape reservoirs with and without Inter-Basin Water Transfer: implications for Sustainable Development Goals

First Reviews	Revision by Author	Second Reviews
**Introduction**		
This is well written but the authors should explain what they mean by Inter-Basin Water Transfer as it is an important variable in the study. Water is used as an entry point to the writing of this manuscript and so it would be important if the status and distribution of water in the areas studied is explained. This is only done briefly by authors explaining in the discussion line 472 to 475.	Answer: Thank you for your observation. We included the definition of IBWT and expanded on this topic to clarify the connection between the benefits and drawbacks of IBWT, landscape changes, ecosystem services, and the SDGs (see lines 73 to 97). Additionally, we have included more details on water distribution in the studied areas within the materials and methods section (see lines 156 to 171). We hope this provides a clearer understanding of the role of water in the context of the study.	The introduction has been re-written and is more clear providing information requested such as explaining the meaning of IBWT
**Materials and methods**		
The authors should consider explaining the role of LULC in the study, is this introduced as a factor impacting the reservoirs? i.e. in line 87, 88, 89 and 90 the author (s) introduces the LULC and states that “The perceptions of riparian communities highlight the essential landscape and ecosystem benefits that are crucial for sustaining life, including water and nutrients, cultural and educational values, and personal environmental experiences”. It is not clear if changes in landscapes impacts water reservoirs which then impact the provisioning of EGS. The authors should consider clarifying.	Answer: Thank you for your valuable comment. We appreciate your attention to this detail. We agree that the use of multiple terms can cause confusion in the text. Therefore, we clarified this point in line 298 of the manuscript and revised the entire text, removing the terms "aquatic environment" and "water body" (lines 299, 437, 438, 461, 581, 585, and 694) to focus on the LULC classification of the reservoir landscapes (“water”), making the text clearer and more accessible for the reader. It is worth mentioning that the reservoir landscapes studied are located in a semi-arid region where it is common for people to construct small or medium-sized dams to store surface water on their properties. These water bodies are part of the reservoir landscapes analyzed.	The initial comment has been addressed<br><br>Remove **add** or check if there is an additional technique missing on line 190<br><br>Line 260 add **to** between conform and research
	Answer: Thank you for the opportunity to clarify this point. While this study did not specifically aim to evaluate changes in LULC and their direct impact on reservoirs and the provision of EGSs, we recognize—based on existing literature—that LULC changes resulting from the implementation of IBWT projects do affect EGS provision. We have made revisions	The methods section on the LULC development has been explained very well

	to the introduction, particularly in lines 73 to 97, to better address this context and clarify the connection. Additionally, we have clarified this point in the Materials and Methods section, on lines (193 to 198). We hope these revisions address the reviewer's concerns.	
Also the author should explain if the study mapped EGS using landscape based approaches and reservoirs was one of the LULC. In short what did the author(s) consider as LULC.<br><br>There is also the interchangeable use of water bodies, reservoirs, aquatic environment . I would advice that the authors stick to this reservoir landscape elements to avoid confusing the readers as there are many variables and treatments in the study and the more different concepts or terms are used interchangeably, the more easier it is to lose a reader. The use of the reservoir landscape elements will also make it easier for the authors to explain the reason for introducing the LULC in the study.	Answer: Thank you for the opportunity to clarify this. We mapped the EGSs based on responses from participatory mapping conducted with the riverside population living within 200 meters of the reservoir, using the reservoir landscape approach. This information was clarified in lines 196 to 198 and line 274. It is important to note that reservoirs, watercourses, and other surface water bodies were classified under the LULC water category, as explained in line 298. We have carefully reviewed these points throughout the manuscript (see lines17, 278, 288, 310, 313, 322, and 352).<br>Additionally, we have renamed the section "Land use and land cover classification" to "Land use and land cover classification: mapping of EGSs and their association with LULC" to make it clearer and more consistent with Figure 2 (see lines 272 to 273).	This questions is addressed adequately
Are interviewees the same as riverside communities in line 566.	Answer: Thank you for your helpful observation. Yes, they are the same. To avoid confusion, we have changed "communities" to "interview participants" in the text and we have restructured the paragraph in the discussion section to make the information clearer for the reader (see lines 621 to 622). Thank you for your observation.	This is corrected
**Discussion and Conclusions**		
These are well written but the sections could be tightened by the authors explaining confounding factor like do reservoirs that receive IBWT and those that are non IBWT reservoirs impact access and water use rights of the local communities and what this mean for the management of the reservoirs	Answer: Thank you for your thoughtful suggestion. We completely agree with your perspective and have addressed this point in the manuscript. We expanded our discussion to explain how reservoirs receiving Inter-Basin Water Transfer (IBWT) and those that are not may affect local communities' access to and use of water, as well as the implications for reservoir management (see lines 642 to 673). Additionally, this perspective was incorporated into the conclusion (see lines 690 to 693).	The discussion covers the issues of access to water and management, giving solutions on management approaches. This is well captured<br><br>Thanks to noting that my input assisted in rethinking your title

	Furthermore, upon reviewing the topic "Influence of LULC on riverside population perceptions of EGSs and the IBWT variable" in the discussion section, we realized that the title was not fully align with our objective and might cause confusion. To improve clarity, we have revised it to "IBWT and the association between perceived EGSs by riverside populations and LULC" (see lines 576 and 577). We hope these changes address your concerns and improve the manuscript's clarity	which is clear and captures the content of your research.

General

Reviewer comment: The manuscript is well written and interesting study.

Answer: Thank you for your dedication in reviewing our work review and positive opinion on our study. We also appreciate your recommendations; they helped improve the quality of our manuscript.

Thank you.
